# Transcriptional Profiles of Diploid Mutant *Apis mellifera* Embryos after Knockout of *csd* by CRISPR/Cas9

**DOI:** 10.3390/insects12080704

**Published:** 2021-08-06

**Authors:** Xiuxiu Wang, Yan Lin, Liqiang Liang, Haiyang Geng, Meng Zhang, Hongyi Nie, Songkun Su

**Affiliations:** 1 College of Life Science, Fujian Agriculture and Forestry University, Fuzhou 350002, China; xiuwang0393@hotmail.com; 2 College of Animal Sciences (College of Bee Science), Fujian Agriculture and Forestry University, Fuzhou 350002, China; ylin19@qub.ac.uk (Y.L.); mifengwang122@163.com (L.L.); haiyang_fafu@163.com (H.G.); ZM_FAFU@163.com (M.Z.); 3 Apicultural Research Institute of Jiangxi Province, Nanchang 330052, China

**Keywords:** *Apis mellifera*, sex determination, CRISPR/cas9, transcriptome

## Abstract

**Simple Summary:**

In honey bees, males are haploid while females are diploid, leading to a fundamental difference in genetic materials between the sexes. In order to better control the comparison of gene expression between males and females, diploid mutant males were generated by knocking out the sex-determining gene, *complementary sex determiner* (*csd*), in fertilized embryos. The diploid mutant drones had male external morphological features, as well as male gonads. RNA sequencing was performed on the diploid mutant embryos and one-day-old larvae. The transcriptome analysis showed that several female-biased genes, such as *worker-enriched antennal* (*Wat*), *vitellogenin* (*Vg*), and some venom-related genes, were down-regulated in the diploid mutant males. In contrast, some male-biased genes, like *takeout* and *apolipophorin-III-like protein (A4)*, were up-regulated. Moreover, the co-expression gene networks suggested that *csd* might interact very closely with *fruitless* (*fru*), *feminizer* (*fem*) might have connections with *hexamerin 70c* (*hex70c*), and *transformer-2* (*tra2*) might play roles with *troponin T* (*TpnT*). Foundational information about the differences in the gene expression caused by sex differentiation was provided in this study. It is believed that this study will pave the ground for further research on the different mechanisms between males and females in honey bees.

**Abstract:**

In honey bees, *complementary sex determiner* (*csd*) is the primary signal of sex determination. Its allelic composition is heterozygous in females, and hemizygous or homozygous in males. To explore the transcriptome differences after sex differentiation between males and females, with genetic differences excluded, *csd* in fertilized embryos was knocked out by CRISPR/Cas9. The diploid mutant males at 24 h, 48 h, 72 h, and 96 h after egg laying (AEL) and the mock-treated females derived from the same fertilized queen were investigated through RNA-seq. Mutations were detected in the target sequence in diploid mutants. The diploid mutant drones had typical male morphological characteristics and gonads. Transcriptome analysis showed that several female-biased genes, such as *worker-enriched antennal* (*Wat*), *vitellogenin* (*Vg*), and some venom-related genes, were down-regulated in the diploid mutant males. In contrast, some male-biased genes, such as *takeout* and *apolipophorin-III-like protein* (*A4*), had higher expressions in the diploid mutant males. Weighted gene co-expression network analysis (WGCNA) indicated that there might be interactions between *csd* and *fruitless* (*fru*), *feminizer* (*fem*) and *hexamerin 70c* (*hex70c*), *transformer-2* (*tra2*) and *troponin T* (*TpnT*). The information provided by this study will benefit further research on the sex dimorphism and development of honey bees and other insects in Hymenoptera.

## 1. Introduction

The queen, the drone, and the worker are the three adults present in a honey bee colony. The queen and drone are responsible for reproduction, while workers mainly participate in colony maintenance, defense, and forage. The queen and workers, two members of the caste system, are females, while drones are adult males. Their behavioral differences correlate with their morphological and physiological differences. They have differences in external morphology (such as eyes, antennae, mouthparts, and hind legs) and internal morphology (like reproductive organs, glands, brain structure, flight musculature, and sensory systems) [1,2,3,4]. Females develop from fertilized eggs that are diploid, while males arise from haploid unfertilized eggs under haplodiploidy. However, males can also be derived from fertilized eggs, usually laid in worker cells [5].

Diploid drones are rare in nature because worker bees eat them within a few hours after hatching [5,6]. Nonetheless, they can be reared to adults under laboratory conditions [7]. In the colonies, nurse bees discover diploid male larvae through the cuticle secretions, in which alkanes are the most significant compounds [8,9]. Moreover, the sex of live first-instar diploid males can be recognized through the contour and size proportions of the epiproct [10]. The discovery of diploid drones enabled the hypothesis of complementary sex determination [11]. The sex-determining gene, *complementary sex determiner* (*csd*), was identified afterward [12]. The allelic composition of *csd* determines the sex of the honey bee. It is heterozygous in females, and hemizygous or homozygous in males [12]. At least five amino acid differences in the potential specifying domain of *csd* can control allelic differences to create femaleness [13]. A total of 116–145 *csd* alleles are presumed to exist in the world [14]. To date, in addition to *csd*, it has been found that *feminizer* (*fem*) [15], *doublesex* (*dsx*) [16], and *transformer-2* (*tra2*) [17] also play a role in the sex-determining pathway of honey bees. As the primary signal, *csd* determines the sex of an individual by controlling the alternative splicing of its downstream gene *fem*, which interacts with *tra2* to trigger the female-specific splicing of *dsx* [17,18].

Haploid (unfertilized) male embryos only appear occasionally with a limited number during the swarming season [19], while fertilized eggs are almost constantly laid. After the swarming season, the males’ population in the colony decreases slowly and dwindles to zero in winter. Haploid males can also synchronously be laid by workers to pass along genes in a queenless colony [20,21]. Diploid males can be obtained in close inbred colonies by artificial insemination of the virgin queen with the semen of its son or brother drones [9]. Diploid males also can be available by knocking out *fem* in fertilized eggs through CRISPR/Cas9 genome editing technology [22]. The CRISPR/Cas9 system has the characteristics of high efficiency, simplicity, and low cost, thus ensuring it is a promising technology for studying gene function. So far, several genes’ functions have been studied in honey bees with this technique [22,23,24,25]. Because the genetic materials between males and females in honey bees are not identical due to the effects of ploidy, using CRISPR/Cas9 to produce diploid mutant males derived from fertilized eggs can better control the comparison of gene expression between males and females.

Here, we knocked out *csd* in fertilized embryos through the CRISPR/Cas9 system. Diploid mutant males were generated with typical male morphological features but with smaller testes than haploid drones. We also explored the transcriptome profiles of diploid mutant males and mock-treated females produced by the same mated queen. Transcripts of the specimens at 24 h, 48 h, 72 h, and 96 h after egg laying (AEL) were analyzed. The results show that some female-biased DEGs were down-regulated, and several male-biased genes had higher expression in diploid mutants. In addition, the co-expression gene networks in which *csd*, *fem*, *tra2,* and *dsx* acted as hub genes were constructed, respectively.

## 2. Materials and Methods

### 2.1. sgRNA Synthesis and Embryo Microinjection

**Figure 1:**
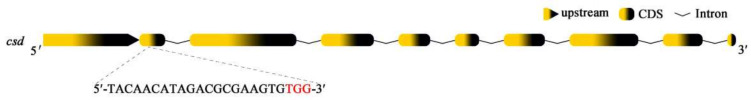
Diagram of the sgRNA targeting site in *csd*. The sgRNA targeting site is in exon 2. The PAM (protospacer adjacent motif) sequence is in red.

The single-guide RNA (sgRNA) for CRISPR/Cas9 was designed on the website http://chopchop.cbu.uib.no/ (accessed on 29 July 2021) [26]. The target site is located in the second exon of *csd* (Figure 1). According to the manufacturer’s instructions, sgRNA was synthesized and purified using an in vitro transcription kit (Inovogen, Chongqing, China). Embryos were harvested from three colonies of Fengqiang No. 1 (*Apis mellifera*), one strain of high royal jelly-producing honey bee [27]. These colonies were maintained in the Apiary of Honey Bee Molecular Breeding Laboratory, Fujian Agriculture and Forestry University, China. After the fertilized queens were caged for one hour on empty combs, the eggs they laid were grafted with a metal needle and fixed on wax strips. Then, the embryos of the treatment groups were microinjected with a mixture of sgRNA and Cas9 nuclease (Invitrogen, Frederick, MD, USA) at a final concentration of 500 ng/µL and a molar ratio of 1:1, using glass capillaries (Narishige, Tokyo, Japan). The eggs of mock-treated groups were injected with sgRNA with a final concentration of 500 ng/µL. All operations were performed skillfully to ensure all the steps, from caging the queen to finishing the microinjection, were completed within two hours. When the embryos were freshly hatched, these young larvae were transferred to Petri dishes containing a modified artificial diet [22], which consisted of 50% royal jelly, 6% glucose, 8% fructose, 2% yeast extract, and 34% water. The condition for the incubation of larvae was 34.5 °C with 90% relative humidity. Additionally, the haploid males laid by the virgin queen were reared with the same ingredients and recipe of food under identical laboratory conditions.

### 2.2. Samples Collection and Mutation Detection

The status of embryos, freshly hatched larvae, and one-day-old larvae were checked using a stereomicroscope (Nikon, Tokyo, Japan), and the live individuals were collected for RNA-seq. The samples were harvested at four time points—24 h, 48 h, 72 h, and 96 h AEL—and three biological replicates were included at each time point. During the collection of samples for RNA-seq, four random samplings were also taken. A total of forty-five 96 h AEL old larvae were collected and raised to adulthood under laboratory conditions. After their eclosion, DNA was extracted. The gene-specific primers (forward primer: 5′-GCGTCTTCTCTAAGCACTT-3′, and reverse primer: 5′-CCACAGTTGTTGTTGTTGAT-3′) were used to amplify the target region. According to the manufacturer’s instructions, Phusion High-Fidelity DNA Polymerase (Invitrogen, Frederick, MD, USA) was used for PCR amplification. The cycling conditions were as follows: denaturation at 98 °C for 3 min, followed by 30 cycles of 98 °C for 30 s, 60 °C for 30 s, and a final extension at 72 °C for 5 min. Then, the genotypes of the target region were examined by TA cloning and sequencing.

### 2.3. Bioinformatic Analysis after RNA-Seq

After extracting total RNA from samples, 24 mRNA libraries were constructed. Then, mRNA sequencing was performed with the Illumina HiSeq 2500 platform (Illumina, San Diego, CA, USA) by the Gene Denovo Biotechnology Company, Guangzhou, China. Raw reads containing adapters or low-quality bases were filtered by fastp (version 0.18.0) [28]. Bowtie2 (version 2.2.8) was used for mapping short reads and clearing short reads [29]. After that, paired-end clean reads were mapped to the reference genome (Amel_HAv3.1) using HISAT (version 2.2.4) [30,31]. Then, the mapped reads were assembled using StringTie (version 1.3.1) [32,33]. For each transcription region, a fragment per kilobase of transcript per million mapped reads (FPKM) value was calculated to quantify its expression abundance and variations [34]. The expressions of differentially expressed genes (DEGs) were analyzed by DESeq2 [35]. The transcripts with the false discovery rate (FDR) <0.05 and absolute fold change ≥2.0 were considered differentially expressed transcripts. To better understand the function of DEGs, they were then subjected to enrichment analysis for Gene Ontology (GO) and KEGG pathways [36,37]. rMATS (version 4.0.1) was used to identify alternative splicing events and analyze differential alternative splicing events between treatment group and mock-treated group samples [38]. Moreover, weighted gene co-expression network analysis (WGCNA) was performed to build gene co-expression networks [39]. After the construction of co-expression modules with the WGCNA package (version 1.47), the modules in which *csd*, *fem*, *tra2*, and *dsx* located were selected. The top 100 genes with the highest weight values in each module were focused and chosen. Also, the DEGs that occurred in these modules, particularly those whose functions were annotated and had high connectivity with *csd*, *fem*, *tra2*, and *dsx* were selected. Additionally, the top 25 genes that showed the highest connectivity with these DEGs were chosen. Co-expression networks, in which *csd*, *dsx*, *fem*, and *tra2* acted as the hub genes, and the selected functional DEGs were respectively built with Cytoscape (version 3.8.1) [40].

### 2.4. qPCR Examination of RNA-Seq Data and Gene Expression in Natural Females and Males

To validate the RNA-seq results’ accuracy, the same samples used for RNA-seq were used for qPCR analysis. Gene-specific primers (Table S1) for the selected genes were employed, and *actin* was the internal reference gene [41]. qPCR was carried out on the CFX384 Touch real-time PCR detection system (Bio-Rad, Hercules, CA, USA). The qPCR kit (GoTaq^®^ qPCR Master Mix, Promega, Madison, WI, USA) was used. Cycling conditions were as follows: denaturation at 95 °C for 3 min, followed by 40 cycles of 95 °C for 15 s, and 60 °C for 20 s. Melting curve analysis was performed at 60 °C for 30 s and 95 °C for 30 s. Relative gene expression levels were calculated using the comparative 2^−ΔΔCt^ method. Statistical significance was estimated through the Student’s *t*-test with SPSS (version 21).

Moreover, natural female samples laid by fertilized queens were collected from the same colonies with mock-treated groups and treatment groups. And, the natural male eggs were laid by the virgin queens subjected to double CO_2_ narcosis [42]. The virgin queens were caged for two hours on empty combs. Same genes’ expression levels were examined in these two natural group samples through qPCR. The primers for each gene, the qPCR verification conditions, and the data analysis methods were identical as those used in the qPCR confirmation of the RNA-seq data.

## 3. Results

### 3.1. Diploid Mutant Males in Which csd Was Knocked by CRISPR/Cas9

**Figure 2:**
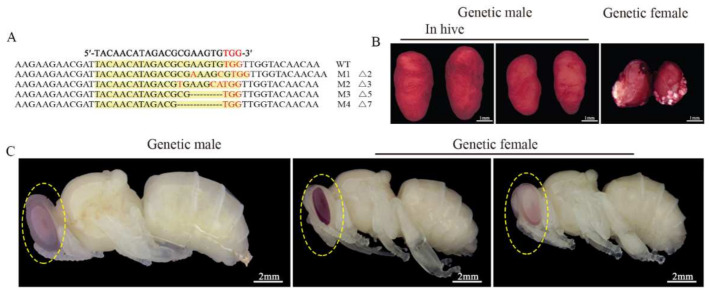
Targeted mutations of csd sequence induced by CRISPR/Cas9 and the morphology of the diploid mutants’ testes. (**A**) Mutated sequences determined by TA cloning and sequencing. The WT sequence is shown at the top. The mutated sequences are located under the WT sequences. Dashes indicate deletions. WT means wild type. M means mutated type. (**B**) The morphology of testes of a diploid mutant drone. The testes on the left are from a freshly emerged drone from a natural colony laid by a fertilized queen. The testes in the middle are from a haploid drone laid by a virgin queen and reared in the laboratory. The testes on the right are from a diploid mutant drone reared under laboratory conditions. 100% (n = 28) of diploid mutant drones had testes. The testes were stained with orcein dissolved with acetic acid. Scale bars, 1 mm. (**C**) The morphology of diploid mutant pupae. All the pupae were reared in the laboratory. The pupa on the left originated from a haploid egg laid by a virgin queen. The pupa in the middle (mock-treated) and the pupa on the right (diploid mutant male) were raised from diploid eggs laid by a mated queen. Scale bars, 2 mm.

**Figure A1:**
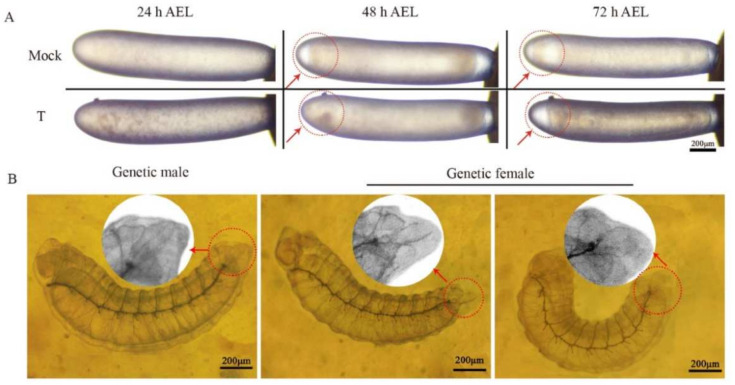
The morphology of the diploid mutant embryos and one-day-old larvae. (**A**) The morphology of the diploid mutant embryos. The eggs of the mock-treated groups were injected with sgRNA. The eggs of the treatment groups were injected with a mixture of sgRNA and Cas9 nucleases. At 24 h AEL, there was no difference in development between the embryos of the mock-treated group and the treatment group. However, obvious immunization responses occurred in the embryos of treatment groups. At 48 h AEL, the treatment group’s embryos were still at stage six, while the mock-treated embryos were at stage seven. At 72 h AEL, the embryos of the treatment group were at stage eight, while the embryos of the mock-treated group were at stage nine. Scale bars, 200 µm. (**B**) The morphology of the epiprocts of the one-day-old diploid mutant larva. The larva on the left was a genetic male; the larvae in the middle and on the right were genetic females. Scale bars, 200 µm.

To collect 80 eggs or 35 larvae used for RNA-seq at each time point, approximately 2300 eggs for each treatment group were injected. For each mock-treated group, about 600 embryos were injected. A total of 28 out of 45 sampling larvae from the treatment groups successfully emerged. They all showed typical morphological characteristics of drones. They all had testes after dissection, which were executed on their first day of adulthood. Then, different mutant genotypes were detected in these diploid mutant drones (Figure 2A). It indicated that the mutation rate of *csd* induced by CRISPR/Cas9 in the treatment group samples used for RNA-seq was 100%. Moreover, according to the honey bee embryos’ stage scheme [43], the development of embryos with treatment was delayed during embryogenesis. At 48 h AEL, the diploid mutant embryos were at stage six, while the mock-treated embryos were at stage seven (Figure A1A). At 72 h AEL, the diploid mutant embryos were at stage eight, while the mock-treated embryos were at stage nine. The newly hatched diploid mutant larvae had a different appearance of epiprocts when compared with the mock-treated larvae (Figure A1B). The diploid mutants showed typical male heads with larger compound eyes during the pupa stage (Figure 2C). Their testes were smaller than the haploid drones derived from the virgin queen and fed with the same artificial diets (Figure 2B).

### 3.2. Evaluation of Transcriptome Data and Gene Expression in Natural Two Sex

**Figure 3:**
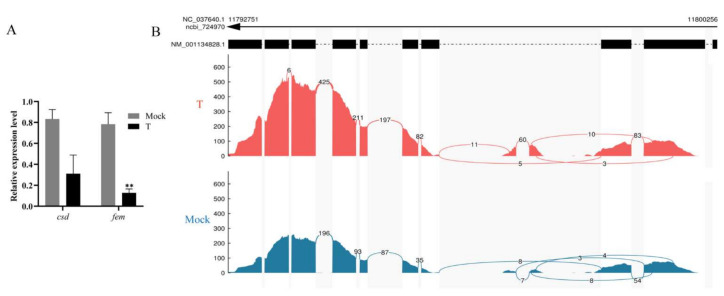
The detection of *fem* splice variants in RNA-seq samples. (**A**) The qPCR examination of the expression of *csd* and *fem^F^* in diploid males. Mock means mock-treated samples injected with sgRNA. T means treatment group specimens injected with the mixture of sgRNA and Cas9 nuclease. The results are given as the mean ± SEM of samples and are expressed as the fold change in mRNA expression. **, *p* < 0.01. (**B**) The reads aligned to each exon of *fem*. More reads aligned to the first four exons in the treatment group samples than those in the mock-treated sample. The reads were from all four time points.

A total of 24 mRNA libraries were established. Over 90.70% of clean reads were mapped to the reference genome in each of them, and over 89.11% of uniquely mapped reads fell within known exons (Table S2). Both *csd* and *fem* were the down-regulated DEGs (FDR < 0.05) at 48 h AEL (Table S3). *Csd* and *fem^F^* in the treatment groups showed significantly lower expression than the mock-treated groups, validated by qPCR (Figure 3A). However, there were more reads of *fem*’s first four exons in diploid mutant specimens than the mock-treated samples in the alternative splicing events analysis (Figure 3B). There is a premature stop codon in the male transcript of *fem* [15]. It is inferred that lots of *fem^M^* transcript isoforms existed in the diploid mutant males.

**Figure A2:**
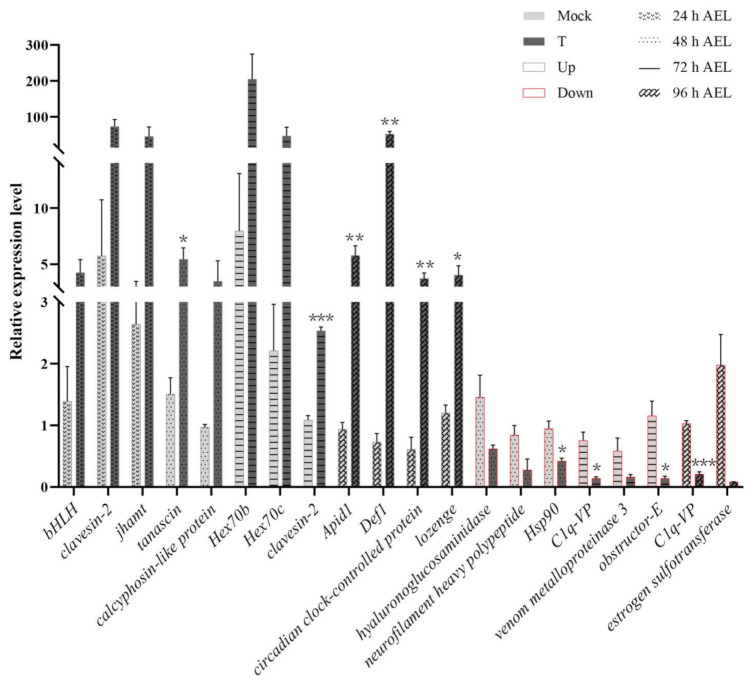
Expression of DEGs from RNA-Seq. Twenty genes were selected and their gene expressions were examined by qPCR. Consistent with RNA-Seq analysis, the up-regulated DEGs were expressed higher in samples of the treatment group than mock-treated specimens; the down-regulated DEGs were expressed higher in specimens of the mock-treated than the treatment group. *Actin* was used as the reference gene. Mock means mock-treated samples injected with sgRNA. T means treatment specimens injected with the mixture of sgRNA and Cas9 nuclease. Up means up-regulated in the treatment group. Down means down-regulated DEGs. The results are given as the mean ± SEM of the samples and are expressed as the fold change in mRNA expression. *, *p* < 0.05; **, *p* < 0.01; ***, *p* < 0.001.

**Figure A3:**
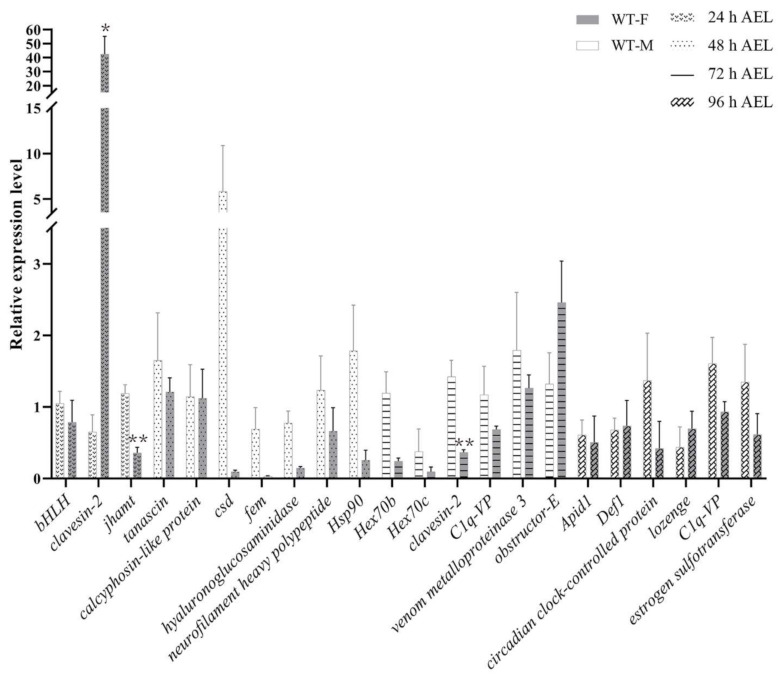
The relative expression level of representative genes was examined in natural females and males. The natural female specimens were taken from the same mother queens, which were shared with the corresponding mock-treated and treatment groups. The natural male eggs were laid by the virgin queens, which were treated with CO_2_ narcosis. The natural female embryos have higher *csd* and *fem* expression levels than natural male eggs. WT-F means natural female. WT-M means natural male. The results are given as the mean ± SEM of samples and are expressed as the fold change in mRNA expression. *, *p* < 0.05; **, *p* < 0.01.

The expression patterns of 20 DEGs selected at four time points were verified through qPCR. The qPCR results show that the expression level of up-regulated DEGs was higher in diploid mutant males; the expression level of down-regulated DEGs was higher in mock-treated females, indicating that the RNA-seq results were credible (Figure A2). Except for *csd* and *fem^F^*, the gene expressions of the same 20 genes were also examined in natural females and males through qPCR. Both *csd* and *fem^F^* had lower relative expression levels in natural male embryos than natural females (Figure A3). In addition, 10 genes showed the same trend with the results of the gene expression level comparison between the mock-treated and the treatment groups.

### 3.3. Analysis of DEGs

**Figure 4:**
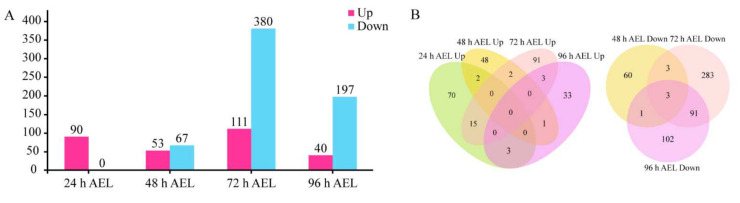
The number of DEGs. (**A**) The number of DEG at four time points. Up means up-regulated DEGs. Down means down-regulated DEGs. (**B**) Venn diagrams of up-regulated and down-regulated DEGs at four time points.

There were 90, 53, 111, and 40 up-regulated and 0, 67, 380, and 197 down-regulated DEGs at 24 h, 48 h, 72 h, and 96 h AEL respectively (Figure 4A). Among the down-regulated DEGs, 94 DEGs were shared at 72 h and 96 h AEL (Figure 4B). According to the existing research [44,45,46,47,48,49,50,51,52,53,54,55,56,57,58,59,60,61,62,63,64,65,66,67,68,69,70,71,72] on these genes’ functions, at 24 h AEL, 13 DEGs were neural-related [44,45,46,47,48,49,50,51,52], and 8 DEGs were associated with muscle [53,54,55] (Table S4). At 48 h AEL, among the up-regulated DEGs, 11 were related to metabolism, 5 were associated with the nervous system [56,57,58,59], and 2 were odorant receptor (OR) encoding genes. The down-regulated DEGs were mainly related to tissue proliferation and functional organs (eye, leg, and wing) [60,61,62] (Table S5). At 72 h AEL, the up-regulated DEGs were involved with the nervous system [44,52], olfactory, and vision [63]. Additionally, four male-biased genes were also up-regulated in diploid mutant males (Table S6) [64,65,66,67]. As for the down-regulated DEGs, some of them were venom-relate genes [68,69,70,71], and several of them were associated with cuticle (epidermis) and ecdysteroid biosynthesis. At 96 h AEL, one up-regulated DEG was vision-related [72]. Most of the down-regulated DEGs were cuticle (epidermis) and ecdysteroid biosynthesis-related (Table S7). There were 21 cuticular protein (CP)-encoding DEGs at 72 h AEL, while the number at 96 h AEL was 14. The down-regulated CP-encoding genes encoded four CP family members: CPR, CPAP3, TWDL, and glycine-rich protein family [73].

### 3.4. GO Enrichment Analyses

**Figure A4:**
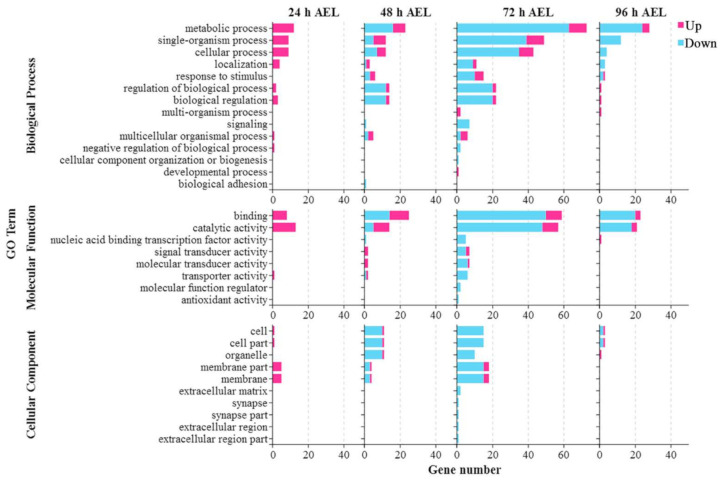
GO analysis of DEGs at four time points. Up means up-regulated DEGs. Down means down-regulated DEGs.

At 24 h AEL, catalytic activity, the metabolic process, the single-organism process, and the cellular process were the top four GO terms that annotated the most DEGs (Figure A4). At 48 h AEL, the up-regulated DEGs were specifically associated with signal transducer activity and molecular transducer activity. In contrast, the GO terms’ enrichment of down-regulated DEGs were uniquely involved in signaling, biological adhesion, and nucleic acid binding transcription factor activity. At 72 h AEL, the up-regulated DEGs were specifically annotated to two GO terms: multi-organism process and developmental process. The down-regulated DEGs were particularly enriched to fifteen GO terms. At 96 h AEL, the up-regulated DEGs were uniquely annotated to five GO terms: regulation of the biological process, biological regulation, multi-organism process, nucleic acid binding transcription factor activity, and organelle. The single-organism process, localization, and the cellular process, specifically, annotated the down-regulated DEGs. The top three GO terms were metabolic process, binding, and catalytic activity, at 48 h, 72 h, and 96 h AEL.

### 3.5. KEGG Pathway Mapping of DEGs

**Figure A5:**
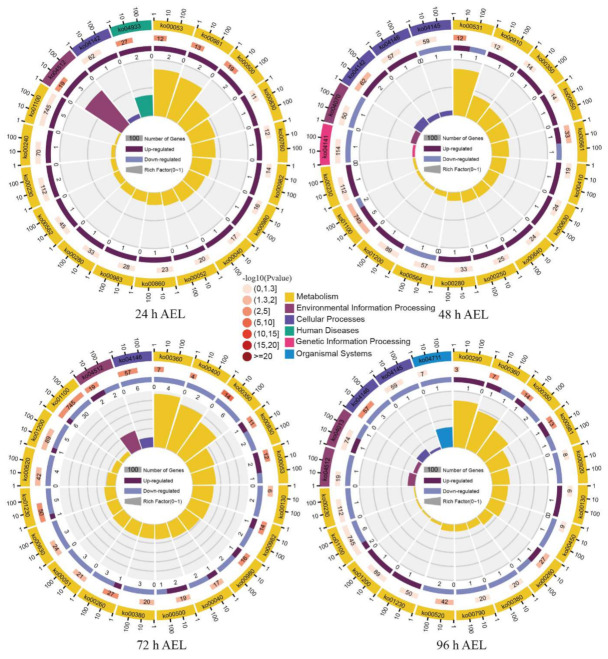
The KEGG pathway enrichment analysis of DEGs. The outermost layer represents the KEGG A class. From outside to inside, the second layer contains the numbers of DEGs with significant enrichment. The third layer shows the up-regulation and down-regulation of the DEGs. The fourth layer is the enrichment factor in each pathway.

At 24 h AEL, the significantly enriched five pathways were the ECM–receptor interaction (ko 04512), human diseases (ko 04933), ascorbate and aldarate metabolism (ko 00053), insect hormone biosynthesis (ko 00981), and starch and sucrose metabolism (ko 00500) (Figure A5). At 48 h AEL, four KEGG pathways, lysosome (ko 004142), glycosaminoglycan degradation (ko 00531), glycerolipid metabolism (ko 00561), and metabolic pathways (ko 01100), were significantly enriched. At 72 h AEL, twenty-two pathways were significantly enriched (Table S8). Among them, the ECM–receptor interaction, ascorbate, and aldarate metabolism were the pathways that were also significantly enriched at 24 h AEL; metabolic pathways were also significantly enriched at 48 h AEL. At 96 h AEL, seven KEGG pathways were significantly enriched. Valine, leucine, and isoleucine biosynthesis (ko00290) was the KEGG pathway uniquely significantly enriched at this time point. Insect hormone biosynthesis (ko 00981) was also significantly enriched at 24 h AEL. Five KEGG pathways, tyrosine metabolism (ko 00350), phenylalanine metabolism (ko 00360), peroxisome (ko 04146), glycine, serine, and threonine metabolism (ko00260), and amino sugar and nucleotide sugar metabolism (ko00520), were also significantly enriched at 72 h AEL.

### 3.6. Co-Expression Network Analysis of csd, fem, tra2, and dsx

**Figure 5:**
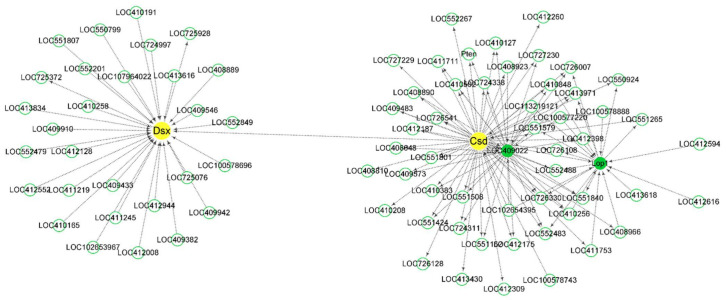
Co-expression network diagram with *csd* and *dsx* acting as the hub genes. *csd* and *dsx* were in the same module and the connectivity between them was very low. The description of LOC409022 on NCBI is *fruitless* (*fru*). Arrows point to downstream genes.

**Figure 6:**
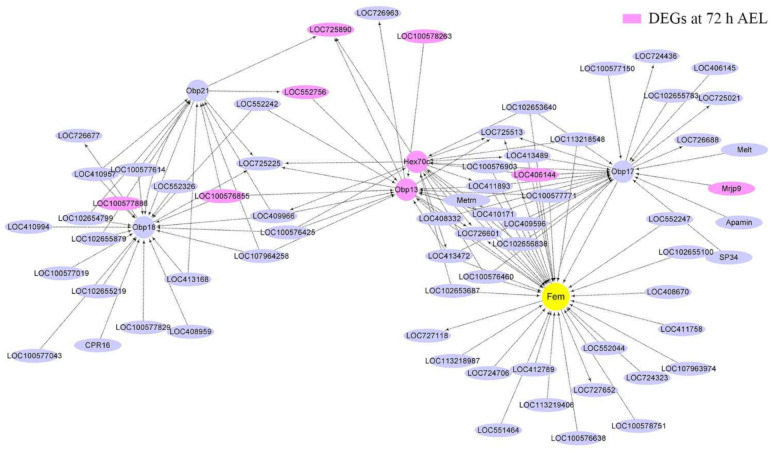
Co-expression network diagram with *fem* acting as the hub gene. A total of 41 up-regulated DEGs at 72 h AEL were in the module *fem* located. Nine of them (background color was fuchsia) are shown in the picture. Arrows point to downstream genes.

**Figure A6:**
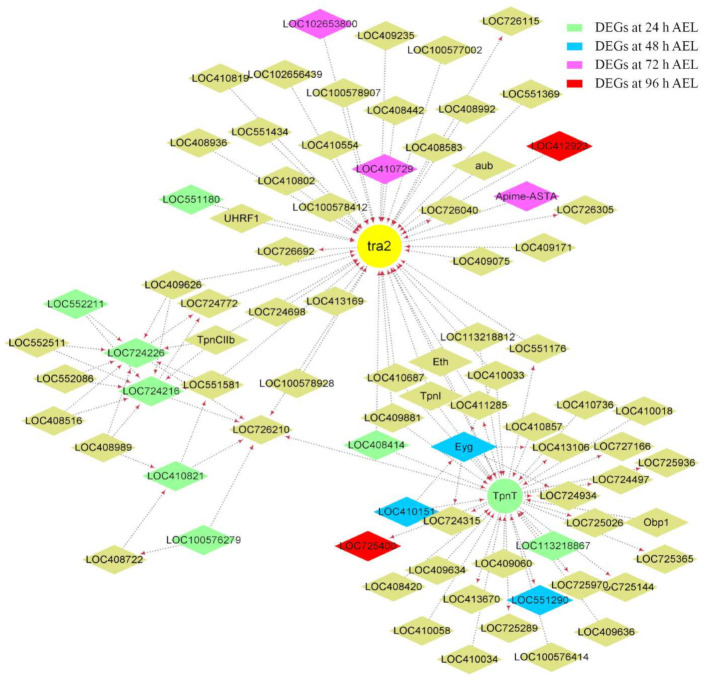
Co-expression network diagram with *tra2* acting as the hub gene. A total of 29, 16, 87, and 24 DEGs were from 24 h, 48 h, 72 h, and 96 h AEL, respectively, also located in this module. Only some of the DEGs that had the top highest weight value with *tra2* are shown in the picture. Their background colors are as follows: pale green, sky blue, fuchsia, and red. Arrows point to downstream genes.

*Csd*, *fem*, *tra2*, and *dsx*, were divided into three modules; *csd* and *dsx* were in the same module. The interaction between *csd* and *dsx* was much more than that between *csd* and *fruitless* (*fru*) (Figure 5). Many genes located downstream of *csd* were also the downstream genes of *fru*. Forty-one up-regulated DEGs at 72 h AEL were in the same module *fem* located. Nine of them were shown in the co-expression networks graph that *fem* acted as the hub gene (Figure 6). Four odorant-binding protein (OBP) encoding genes, *Obp13*, *Obp17*, *Obp18*, and *Obp21*, were also included in the module. *Obp13* was an up-regulated DEG at 72 h AEL. The co-expression networks showed that *Obp13* and *Obp17* had more intense relationships with *fem* than *Obp18* and *Obp21*. *Obp13*, *Obp17*, and *hexamerin 70c* (*hex70c*) were the upstream genes of *fem*. A total of 29, 16, 87, and 24 DEGs belonging to 24 h, 48 h, 72 h, and 96 h AEL, respectively, were in the module *tra2*. There were six *troponin* (*Tpn*) family members, *TpnII*, *TpnCI*, *TpnC IIa*, *TpnCIIb*, *TpnCIIIa,* and *TpnT*. *tra2* seemed to have an intense interaction with the *Tpn* family, especially *TpnT*, an up-regulated DEG at 24 h AEL (Figure A6).

## 4. Discussion

After *fem* was knocked out in honey bees through CRISPR/Cas9 to generate the diploid mutant males [22], *csd* was knocked out with the same technique in the present study, and the diploid mutant males were obtained. The natural haploid and diploid male eggs require about three hours longer than female eggs to hatch [4,74]. In this study, the development of the diploid mutant male embryos was delayed when compared with the mock-treated embryos (female). The haploid and diploid male first-instar larvae showed no difference in the contour of epiproct [10]. The diploid mutant larvae had different epiproct characters compared with their female sisters in this study. Moreover, the testes of the diploid adult mutants were smaller than the testes of the haploid drones fed with the same ingredient and recipe diets. In previous studies, in *Apis mellifera*, the diploid drones’ testes were smaller than those of the haploids’ when they were killed on their first day of adult life [9,75]. This is mainly because of the fewer and shorter testicular tubules in the testes of diploid males than the normal testes [76]. Similar situations happened in the newly emerged diploid drones of *Apis cerana*, which had a lighter wet weight of reproductive organs than the haploid drones [77]. In addition, during the larval stage, the testes of diploid fifth-instar larvae were smaller than normal ones when *fem* was knocked down [18]. However, the diploid male larvae’ testes had full-sized development when *csd* was knocked down [18] and when *fem* was knocked out [22].

So far, several omics studies comparing natural males and females (Table S9), which mainly were transcriptome [78,79,80,81] and proteome [82,83], have been carried out. A transcriptome comparison was also conducted between haploid drones and diploid drones in *Apis cerana* [77]. These studies covered the main stages during the honey bee life cycle: embryogenesis [78], larval stage [79], pupal stage [80], and adult life [77,81,82,83]. Additionally, a comparative study of the transcriptome at different time points in the embryonic period in *Apis cerana* was also conducted [84]. A comparison of the results in the present study with the findings in previous studies was executed.

At 24 h AEL, a large percentage of DEGs were neural-related, consistent with the KEGG pathway—extracellular matrix (ECM)—was significantly enriched. ECM plays a fundamental role in the nervous system’s development, maintenance, and regeneration [85]. The insect hormone biosynthesis pathway was also significantly enriched. *Juvenile hormone acid O-methyl transferase* (*jhamt*) was the DEG that participated in this pathway. The protein *jhamt* encoded is an essential enzyme in the JH biosynthesis pathway in insects [86]. In honey bees, queens have higher expression levels of *jhamt* than workers in almost all developmental stages [87]. In the present study, the natural male embryos had a lower mRNA expression of *jhamt* than natural females at 24 h AEL. However, its expression was higher in diploid mutant males than the mock-treated females. JH affects the nervous system structure in adult honey bees [88]. It was presumed that the differentiation between males and females might begin with neural fate determination which influenced by *jhamt* after the sex differentiation. Moreover, the number of significantly enriched GO terms annotated by *ryanodine receptor* (*RyR*) was the largest. Five of them were involved in the biological process category, and three GO terms were tied to the cellular component category. *RyR* encoded a Ca^2+^ release channel family in *Drosophila* [51]. The gene expression level of *RyR* and six other DEGs (*thrombospondin type-1 domain-containing protein 4*, *nidogen-2*, *synaptic vesicle glycoprotein 2B*, *neural cell adhesion molecule 2*, *neuroligin 1*, and *omega-conotoxin-like protein 1*) was higher in the antennae of foragers than those in drones [81]. Furthermore, *takeout*, regulated by the somatic sex-determination pathway and affecting male courtship behavior [89], was also up-regulated in diploid mutant embryos.

The role of males playing in the colony is to mate with virgin queens, which relies heavily on the olfactory detection of the queens’ pheromones. The realization of this function is inseparable from the antennae of the drone. Except for apparent differences in drone and worker antennae’s shape and size, the most dramatic difference between them lies in the number of pore plate sensilla, which makes drones possess as many as five times olfactory sensory neurons as workers [90]. At 48 h AEL, two olfactory-related genes, *Or4–like* and *Or35*, were up-regulated in diploid mutant embryos. Four GO terms, annotated by the former, were significantly enriched. One belonged to the molecular function category, and the other three belonged to the biological process category. As for the GO terms that *Or35* annotated, two of them were significantly enriched. They were classified into the biological process category and the molecular function category. *Or4-like* was also an up-regulated DEG at 72 h AEL. Other olfactory-related DEGs, *Or4*, *Or13a*, *Obp13*, and *Obp14*, were also up-regulated at this time point. *Obp13* was one of the genes controlled by sex-determination pathways and caste signals [80]. The protein encoded by *Obp14* had an exclusive up-regulation in the antennae of drones [82]. None of these olfactory-related DEGs were up-regulated in the antennae of foragers [81]. As for the down-regulated DEG at 48 h AEL, fourteen GO terms from three categories annotated by *eyg*, one Notch pathway gene, were significantly enriched. *Eyg* promoted eye–antennal disc proliferation and controlled the eyes’ growth in *Drosophila* [60,61,62]. Twelve GO terms annotated by *forkhead domain transcription factor slp1* were significantly enriched. Five of them were from the biological process category, four were classified to the molecular function category, and three belonged to the molecular function category. *Forkhead domain transcription factor slp1* is responsible for the development and differentiation of *Drosophila* [91]. It was inferred that the down-regulation of these development-related DEGs might have connections with the delayed development of diploid mutant embryos.

At 72 h AEL, eight down-regulated DEGs (*worker-enriched antennal* (*Wat*), *venom serine protease Bi-VSP*, *venom carboxylesterase-6-like*, *venom dipeptidyl peptidase 4-like*, *C1q-like venom protein* (*C1q-VP*), *venom acid phosphatase Acph-1-like protein* (*Acph-1*), *nuclear hormone receptor Ftz-f1*, and *loricrin*) showed higher expressions in the antennae of foragers than drones [81]. *Wat* was controlled by the combined action of the sex determination and the caste pathways [80]. *Venom carboxylesterase-6-like* was also found to have significant up-regulation in queen larvae compared with drone larvae [79]. The venom glands were evolved from the female accessory reproductive glands and were consequently restricted to female bees [92]. The expression profile of *C1q-VP* was lower in natural males and diploid mutant males when compared with females at 72 h and 96 h AEL, respectively. The down-regulation of venom-related genes in diploid mutant males might be due to sex change. In addition, *apolipophorin-III-like protein* (*A4*) was also one of the up-regulated DEGs at 72 h AEL. *A4* was uniquely isolated in the antennal of the male fire ant, *Solenopsis invicta* [65]. Moreover, *cationic amino acid transporter 3* (*CAT*), which activates *vitellogenin* (*Vg*) [93], was also down-regulated in diploid mutant males at 72 h AEL. *Vg* was the down-regulated DEG at AEL 96 h. *Vg* encodes a female-specific protein in honey bees [94] and performs many functions in females [95,96]. In previous research, the expression of *Vg* was higher in forager antennae than males’ [81]. Vg’s concentration in haploid drones was low and could only be produced a few days after emergence [94,97]. The overage concentration of Vg in diploid males was only about half of that of the haploid drones [9]. Furthermore, the expressions of *tyrosine hydroxylase* and *tyrosine aminotransferase* were lower in natural drones [81] They were the down-regulated DEGs at 96 h AEL in the present study. The tyrosine metabolism pathway they participated in was also significantly enriched.

WGCNA results suggest that *csd* has a remoter relationship with *dsx*, which is consistent with *dsx* acting as a bottom gene in the sex-determination pathway. *Fru* is a neuronal gene that acts to establish the male courtship behaviors in *Drosophila* [98,99,100]. In the module in which *fem* acted as the hub gene, *fem* was a downstream gene of *hex70c*. *Hex70c* encodes a storage protein whose expression was strongly and positively influenced by JH [64]. It was speculated that *fem* might also be affected by JH. *Tra2* has been proved to have a vital role in honey bee embryogenesis [17]. The co-expression network showed that *TpnT*, an up-regulated DEG at 24 h AEL, had an intense interaction with *tra2*. The other five *Tpn* family members, *TpnI*, *TpnCI*, *TpnCIIa*, *TpnCIIb*, and *TpnCIIIa*, were also in the same module. *TpnT*, *TpnI*, and *TpnCIIb* were co-regulated by the sexual and caste signal [80]. Four GO terms annotated by *TpnT* were significantly enriched. *TpnT* could affect the development of indirect flight muscles in *Drosophila* [53]. The troponin complex was considered a vital role in regulating the contraction and relaxation of striated muscles [54]. It could be inferred that *tra2* might participate in the development of striated muscles in honey bees with the company of *TpnT*.

## 5. Conclusions

In this study, diploid mutant males were generated by knocking out *csd* in fertilized eggs laid by a mated queen. The morphology of diploid mutant drones had typical male characteristics with smaller testes. The transcriptome analysis of diploid mutant males and mock-treated females that shared the same mother queen showed that some female-biased DEGs were down-regulated, while several male-biased genes were up-regulated in diploid mutant males. Among the DEGs at 24 h AEL, a large percentage of DEGs were neural-related. At 48 h and 72 h AEL, several up-regulated DEGs were olfactory-related, which might have a connection with the different roles of males and females played in colonies. The down-regulation of development and differentiation-related DEGs at 48 h AEL, as well as cuticle (epidermis) and ecdysteroid biosynthesis-related DEGs at 72 h AEL, might connect with the delayed development of diploid mutant embryos. Additionally, *fru* might also take part in the sex-determining pathway, and as the downstream gene of *csd*. *fem* might have interactions with *Obp13*, *Obp17,* and *hex70c*. Furthermore, the *Tpn* family, especially *TpnT*, might join with *tra2* to participate in embryos’ development. Basic information about the gene expression of females and diploid mutant males was shown in this study. More validation work is needed to discover their mysterious roles in honey bees. Particularly, the genes related to sex dimorphism are valuable for further gene function study.

## Data Availability

The data presented in this study are available on request from the first author.

## Supplementary Materials

The following are available online at https://www.mdpi.com/article/10.3390/insects12080704/s1, Table S1: Gene-specific primers for qPCR; Table S2: The quality of RNA-seq data after mapping to the reference genome of *Apis mellifera*; Table S3: The transcripts of *csd* and *fem* at four time points; Table S4: Nerve- and muscle-related DEGs at 24 h AEL; Table S5: DEGs related to the development of tissues and organs at 48 h AEL; Table S6: The main DEGs at 72 h AEL; Table S7: The main DEGs at 96 h AEL; Table S8: The significantly enriched KEGG pathways at 72 h AEL; Table S9: The omics research about the comparison of male and female in honey bees.
