# Supplementary material for: Transcriptional Profiles of Diploid Mutant Apis mellifera Embryos after Knockout of csd by CRISPR/Cas9
Source: Insects. 2021 Aug 6;12(8):704. doi: 10.3390/insects12080704 (PMC8396534; doi:10.3390/insects12080704)
Supplement: Supplementary file 1 [file insects-12-00704-s001.zip › insects-1316603-supplementary.pdf]

insects MDPI

Table S1. Gene-specific primers for qPCR.

Symbol	Description	Primer
Arp1	actin related protein 1	5′-TGCCAACACTGTCCTTTCTG-3′<br>5′-AGAATTGACCCACCAATCCA-3′
Csd	complementary sex determiner	5′-ACAAGTCGTAAGCGTTATTC-3′<br>5′-ATAGGAACAGGAACAGGAAC-3′
Fem	feminizer	5′-TTCAGCAGAACTCGTCAA-3′<br>5′-CTTCCAACGGTGACTCTC-3′
LOC100576700	class A basic helix-loop-helix protein 15 (bHLH)	5′-ATTCCCTAAATGACGCTTTC-3′<br>5′-GCAGACTGTTATTGTTGGTT-3′
LOC552447	clavesin-2	5′-GTTCTATCTGATCCACGAGTT-3′<br>5′-TTCACCGCCATAATTCTCC-3′
LOC724216	juvenile hormone acid O-methyltransferase (jhamt)	5′-GAAGAGATGTCCGAGATGAA-3′<br>5′-CTCCTGGTCGCAACAATT-3′
LOC100577875	tenascin	5′-GGACACATTGGGATATGGA-3′<br>5′-CGATCACCTATCATCAATGG-3′
LOC409367	calcyphosin-like protein	5′-CGCAACAACTAGGCAAGA-3′<br>5′-AACTCATCGACGCTAATGT-3′
LOC406146	hyaluronoglucosaminidase	5′-ACTGTCCGTAGAGGTGGTTC-3′<br>5′-TTCGCCGCTCGTCAGATT-3′
LOC725960	neurofilament heavy ploypeptide	5′-TAGCGATGGTGTGCCTGTT-3′<br>5′-AGCCTCCTCCTTCTTCTCCT-3′
Hsp90	heat shock protein 90	5′-AATCTTCTGCTGGTGGTTCTT-3′<br>5′-TCATCTCCTCCAACTTCTTCAA-3′
Hex70b	hexamerin 70b	5′-GGCAGAAGAACATCTACGAACT-3′<br>5′-ATCACGGCTACGCTTAACG-3′
Hex70c	hexamerin 70c	5′-GCACACCGCCGATATGGAT-3′<br>5′-GCCGCTGTTCATCCGAAGT-3′
C1q-VP	C1q-like venom protein	5′-ATGGTGGTGTGGCTAGTGTT-3′<br>5′-TTAATCGTAGATCGGTGCTTCC-3′
LOC409468	venom metalloproteinase 3	5′-CTTGTGGAGACGGTCAGTATTG-3′<br>5′-ACTTGCTGTGGTGTAGGTGTA-3′
LOC100577576	protein obstructor-E	5′-GTCCCGAGCCCAAAGGTTT-3′<br>5′-ACGCCGTCTATGCAGTTCA-3′
Apid1	apidaecin 1	5′-CCGTGAAGCTGAACCGAAAG-3′<br>5′-AGGTGGTCTTGGTTGTGGAA-3′
Def1	defensin 1	5′-CCTTCTCTTCATGGCTATGGTT-3′<br>5′-CAAACTCCTTTCTCGCAATGAC-3′
LOC727010	circadian clock-controlled protein	5′-CGTGGATGGACAACTACTG-3′<br>5′-GTTCTCGATGATAGGCGTTA-3′
LOC410537	protein lozenge	5′-GGAAGAAGTGGTAGAGGCAAGA-3′<br>5′-CAGGCACCGCTAACCGAAT-3′
LOC412996	estrogen sulfotransferase	5′-TCAATCATCCTGAGGTAACGC-3′<br>5′-GCTTCCTTCAAATGTTCCCAAT-3′

Table S2. The quality of RNA-seq data after mapping to the reference genome of *Apis mellifera.*

Sample	RawData	CleanData (%)	Total	Unique_Mapped (%)	Total_Mapped (%)	AF_Q30 (%)
Mock24 h-1	45133220	45054638 (99.83%)	38414402	35277586 (91.83%)	35691777 (92.91%)	6280491691 (93.50%)
Mock24 h-2	51963068	51887650 (99.85%)	45472432	42909198 (94.36%)	43477745 (95.61%)	7128774144 (92.06%)
Mock24 h-3	40364952	40316470 (99.88%)	38612558	36862689 (95.47%)	37200716 (96.34%)	5568701877 (92.85%)
Mock48 h-1	42676328	42632456 (99.90%)	31076436	29267015 (94.18%)	29710205 (95.60%)	5920667192 (93.47%)
Mock48 h-2	48363816	48296550 (99.86%)	43517214	40952301 (94.11%)	41581823 (95.55%)	6668394368 (92.50%)
Mock48 h-3	41512114	41463952 (99.88%)	33820466	32003228 (94.63%)	32409714 (95.83%)	5748789473 (92.81%)
Mock72 h-1	48780040	48708850 (99.85%)	43473122	40771002 (93.78%)	41557103 (95.59%)	6805089627 (93.85%)
Mock72 h-2	41521280	41463470 (99.86%)	35209940	32921693 (93.50%)	33415318 (94.90%)	5714944808 (92.45%)
Mock72 h-3	42380676	42329862 (99.88%)	35318354	33207585 (94.02%)	33794901 (95.69%)	5861676447 (92.78%)
Mock96 h-1	51484852	51407908 (99.85%)	42446068	39996056 (94.23%)	40734105 (95.97%)	7176710739 (93.94%)
Mock96 h-2	47234124	47161810 (99.85%)	41144244	39058538 (94.93%)	39752087 (96.62%)	6638774383 (94.30%)
Mock96 h-3	42678804	42625394 (99.87%)	37942452	36011318 (94.91%)	36552783 (96.34%)	5905655162 (92.79%)
T24 h-1	43213308	43159572 (99.88%)	39035594	36639834 (93.86%)	37110663 (95.07%)	5989861118 (93.16%)
T24 h-2	35548604	35496288 (99.85%)	29302992	27475263 (93.76%)	27799123 (94.87%)	4903162460 (92.51%)
T24 h-3	44617220	44569524 (99.89%)	40718006	38872826 (95.47%)	39276681 (96.46%)	6217932182 (93.70%)
T48 h-1	45365032	45311744 (99.88%)	38570206	36397979 (94.37%)	36912999 (95.70%)	6300242644 (93.33%)
T48 h-2	50709892	50641034 (99.86%)	45487822	43010699 (94.55%)	43665781 (95.99%)	6995734062 (92.53%)
T48 h-3	46175096	46123954 (99.89%)	39583860	37522461 (94.79%)	38052823 (96.13%)	6406022029 (93.00%)
T72 h-1	69199710	69105948 (99.86%)	63769012	60151690 (94.33%)	61082070 (95.79%)	9672468024 (93.90%)
T72 h-2	37915378	37860176 (99.85%)	33709480	31659055 (93.92%)	32104388 (95.24%)	5226371633 (92.64%)
T72 h-3	46409018	46342504 (99.86%)	42233560	40015871 (94.75%)	40528080 (95.96%)	6411290682 (92.68%)
T96 h-1	46361444	46291730 (99.85%)	40968078	38652791 (94.35%)	39385637 (96.14%)	6478096918 (93.80%)
T96 h-2	47828266	47763436 (99.86%)	44414968	39579678 (89.11%)	40282920 (90.70%)	6674170314 (93.99%)
T96 h-3	43658676	43605272 (99.88%)	41505676	39412716 (94.96%)	40012249 (96.40%)	6040717052 (92.90%)

Table S3. The transcripts of *csd* and *fem* at four-time points.

Id	Symbol	Description	Time point	log2(fc)	*p*-Value	FDR
ncbi_406074	Csd	complementary sex determiner	24 h AEL	−3.4250745	0.03208526	0.999979
			48 h AEL	−2.2735837	0.0000351	0.007498
			72 h AEL	−0.8211519	0.12779848	0.958579
			96 h AEL	0.53666694	0.813445059	0.999995
ncbi_724970	Fem	feminizer	24 h AEL	−2.0909322	0.000215512	0.085075
			48 h AEL	−1.5425479	0.00000944	0.004215
			72 h AEL	0.52795826	0.807475153	0.999955
			96 h AEL	1.55355153	0.001538592	0.061052

Table S4. Nerve- and muscle-related DEGs at 24 h AEL.

Symbol	Description	Function
LOC100576700	class A basic helix-loop-helix protein 15, transcript variant X1	neurogenesis
LOC408579	thrombospondin type-1 domain-containing protein 4	neuronal development
LOC408797	nidogen-2	nervous system patterning
LOC409924	synaptic vesicle glycoprotein 2B	neurotransmission
LOC410380	neprilysin-2, transcript variant X2	regulation of neuropeptide signaling
LOC410821	tetraspanin-9	nervous system, cell signaling
LOC410824	spondin-1, transcript variant X1	axon extension
LOC411212	discoidin domain containing receptor2	nonintegrin collagen receptor
LOC552447	clavesin-2	neuron-specific membrane-associated protein
LOC552513	neural cell adhesion molecule 2, transcript variant X1	synaptic plasticity
LOC724358	neuroligin 1, transcript variant X1	synapse development
LOC408680	ryanodine receptor, transcript variant X1	calcium release
LOC725074	omega-conotoxin-like protein 1	calcium channel in neurons
LOC113218867	paramyosin, short form-like	muscle associated
LOC408414	tropomyosin-1	formation of germ
LOC409787	paramyosin, long form	flight muscle
LOC410058	myosin light chain alkali	flight muscle
LOC410204	titin, transcript variant X1	myofibrillar components
LOC551259	titin, transcript variant X1	myofibrillar components
TpnT	troponin T, skeletal muscle	myofibrilogenesis
TpnC Ⅰ	troponin C type Ⅰ	troponin family member
LOC724216	juvenile hormone acid O-methyltransferase	JH biosynthesis pathway

Table S5. DEGs related to the development of tissues and organs at 48 h AEL.

Expression Trend	Symbol	Description	Function
Up-regulated	Tk	tachykinins	intrinsic neuroprotective
	LOC413558	photoreceptor-specific nuclear receptor (PSNR), transcript variant X1	neurotransmitters in specific interneurons
	tβh	tyramine β-hydroxylase	neurotransmitter
	LOC409957	synaptotagmin 4 (syt4), transcript variant X1	neuronal Ca^{2+} sensor
	LOC408955	4-aminobutyrate aminotransferase, mitochondrial, transcript variant X3	neurotransmitter
Up-regulated	LOC100577101	odorant receptor 4-like	odorant receptor encoding gene
	Or35	odorant receptor 35	
Up-regulated	LOC100576484	caspase-1, transcript variant X1	
	LOC408955	4-aminobutyrate aminotransferase, mitochondrial, transcript variant X3	
	LOC410148	carboxypeptidase Q	
	LOC410733	glucose dehydrogenase, transcript variant X3	
	LOC411353	lipase 3	
	LOC412007	facilitated trehalose transporter Tret1, transcript variant X3	
	LOC551437	alpha-N-acetylglusaminidase, transcript variant X2	
	LOC552357	maltase A3, transcript variant X2	
	LOC552771	hydroxyacid oxidase1, transcript variant X1	
	LOC726880	pancreatic lipase-related protein 2-like	
	LOC727237	carbonic anhydrase 2	
Down-regulated	LOC552546	protocadherin Fat 4, transcript variant X1	planar cell polarity, oriented cell division
	LOC726165	fork head domain transcription factor slp1	development and differentiation
	LOC102656170	protein atonal (ato)	cellular proliferation to tissue differentiation
	LOC100577069	hairy/enhancer-of-split (HES)	cellular proliferation to tissue differentiation
Down-regulated	Eyg	Eyegone	eye growth
	LOC410853	irregular chiasm C-roughest (IrreC-rst)	target recognition in the optic neuropils
	LOC410151	retinal homeobox protein Rx2, transcript variant X1	establishment of the visual system
Down-regulated	LOC100577692	transcription button head (btd), transcript variant X1	fundamental role in arthropod leg development
Down-regulated	LOC726247	protein vestigial transcript variant X1	wing
Down-regulated	LOC406146	hyaluronoglucosaminidase (HYAL)	basic glycoprotein of the venom's dry matter

Table S6. The main DEGs at 72 h AEL.

Expression Trend	Symbol	Description	Function
Up-regulated	LOC100576700	class A basic helix-loop-helix protein 15, transcript variant X1	neurogenesis
	LOC409924	synaptic vesicle glycoprotein 2B, transcript variant X1	neurotransmission
	LOC411186	synaptic vesicle glycoprotein 2C, transcript variant X1	neurotransmission
Up-regulated	Hex70b	hexamerin 70b	transcripts abundant in the larval gonads of drones
	A4	apolipophorin-III-like protein	isolated in the antennal of the male fire ant
	LOC113218865	dynein heavy chains1, axonemal-like	providing the force for the beating of flagella and cilia
	LOC725593	dynein heavy chains7, axonemal, transcript variant X1	providing the force for the beating of flagella and cilia
Up-regulated	LOC100576816	odorant receptor 4, transcript variant X1	
	LOC100576522	odorant receptor 13a, transcript variant X1	
	Obp13	odorant binding protein 13	
	Obp14	odorant binding protein 14	
Up-regulated	LOC413168	retinol dehydrogenase (RDH) 14	
	LOC100576816	retinol-binding protein pinta-like, transcript variant X1	
Up-regulated	Apid1	apidaecin1	antimicrobial peptide
	LOC406142	hymenoptaecin	antibacterial
	LOC406144	abaecin	antibacterial
	LOC725225	ninjurin-2	prevent inflammatory disease
Down-regulated	Wat	worker-enriched antennal transcript	
	LOC551232	probable nuclear hormone receptor HR 38	
	LOC409646	cationic amino acid transporter 3, transcript variant X1	activates the vitellogenin
Down-regulated	LOC409143	venom serine protease 34, transcript variant X2	
	LOC410526	serine protease 42, transcript variant X3	presented in bee venom
	LOC724308	serine protease 53	presented in bee venom
	LOC408395	venom carboxylesterase-6-like, transcript variant X2	
	LOC409468	venom metalloproteinase 3, transcript variant X2	
	LOC550671	venom serine protease Bi-VSP	
	LOC408395	venom carboxylesterase-6-like	
	LOC411229	venom dipeptidyl peptidase 4-like, transcript variant X1	
	C1q-VP	C1q-like venom protein	
	LOC724418	venom acid phosphatase Acph-1-like protein (Acph-1)	
Down-regulated	LOC410365	laccase2 (Amlac2)	exoskeleton differentiation
	LOC409962	loricrin	major protein component of the cornified cell
Down-regulated	LOC726450	nuclear hormone receptor FTZ-F1	ecdysteroid biosynthesis
	LOC551632	methyl farnesoate epoxidase (mfe), transcript variant X1	JH biosynthesis
	LOC724386	niemann–Pick type C2 (NPC2) intracellular cholesterol transporter 2	ecdysteroid
Down-regulated	LOC724464	cuticular protein	
	CRP1	cuticular protein 1	
	CRP2	cuticular protein 2	

CRP3	cuticular protein 3
CRP4	cuticular protein 4
CRP6	cuticular protein 6
CRP11	cuticular protein 11
CRP12	cuticular protein 12
CRP13	cuticular protein 13
CRP14	cuticular protein 14
LOC726451	cuticular protein 16.8
CRP17	cuticular protein 17
CRP18	cuticular protein 18
CRP21	cuticular protein 21
CRP22	cuticular protein 22
CRP27	cuticular protein 27
Grp	glycine-rich cuticle protein
LOC724398	tweedle motif cuticular protein2
LOC107965773	larval cuticle protein LCP-17, transcript variant X1
LOC100576182	pupal cuticle protein G1A
LOC113218932	pupal cuticle protein

Table S7. The main DEGs at 96 h AEL.

Expression Trend	Symbol	Description	Function
Up-regulated	LOC100577142	G-protein coupled receptor methuselah (Mth)	
	Apid1	apidaecin1	Antimicrobial peptide
	Def1	Defensin1	antimicrobial peptide
	LOC552672	proclotting enzyme	immune
Up-regulated	LOC410537	protein lozenge (LZ)	visual system
Down-regulated	LOC410526	serine protease 42, transcript variant X3	presented in bee venom
	C1q-VP	C1q-like venom protein	
	LOC724418	venom acid phosphatase Acph-1-like protein (Acph-1)	
Down-regulated	Vg	vitellogenin	female specific protein
Down-regulated	LOC409002	cuticular protein	
	LOC724464	cuticular protein	
	LOC725089	cuticular protein	
	CRP1	cuticular protein 1	
	CRP2	cuticular protein 2	
	CRP4	cuticular protein 4	
	CRP6	cuticular protein 6	
	CRP10	cuticular protein 10	
	CRP11	cuticular protein 11	
	CRP17	cuticular protein 17	
	CRP27	cuticular protein 27	
	CPF1	cuticular protein CPF1	
	LOC107965773	larval cuticle protein LCP-17, transcript variant X1	
	LOC726950	pupal cuticle protein 20, transcript variant X1	

Table S8. The significantly enriched KEGG pathways at 72 h AEL.

Pathway	Mock vs. T (70)	All (2429)	*p*-Value	Pathway ID
Tyrosine metabolism	6	14	1.15647E-06	ko00350
Phenylalanine metabolism	4	7	2.07634E-05	ko00360
Metabolic pathways	36	745	0.000189225	ko01100
ECM-receptor interaction	4	19	0.001768037	ko04512
Biosynthesis of amino acids	6	50	0.002692802	ko01230
Retinol metabolism	3	11	0.003204472	ko00830
Ascorbate and aldarate metabolism	3	12	0.004184917	ko00053
Phenylalanine, tyrosine and tryptophan biosynthesis	2	4	0.004732185	ko00400
Peroxisome	6	57	0.005246194	ko04146
Drug metabolism cytochrome P450	3	14	0.006643254	ko00982
Glycine, serine and threonine metabolism	4	27	0.006726895	ko00260
Metabolism of xenobiotics by cytochrome P450	3	16	0.009806765	ko00980
Pentose and glucuronate interconversions	3	17	0.0116652	ko00040
Starch and sucrose metabolism	3	19	0.01595258	ko00500
Tryptophan metabolism	3	20	0.01838615	ko00380
Fructose and mannose metabolism	3	21	0.0210149	ko00051
Ubiquinone and other terpenoid-quinone biosynthesis	2	9	0.02585921	ko00130
Glyoxylate and dicarboxylate metabolism	3	24	0.03007629	ko00630
Amino sugar and nucleotide sugar metabolism	4	42	0.03104666	ko00520
Carbon metabolism	6	89	0.04082755	ko01200
Nicotinate and nicotinamide metabolism	2	12	0.04484458	ko00760
Drug metabolism—other enzymes	3	28	0.04487149	ko00983

Table S9. The omics researches about the comparison of male and female in honey bees.

Stage	Timing	Organ	Male	Female	Number of libraries	omics	Subspecies	Citation
embryos	0–2 h, 0–6 h, 18–24 h	mature oocytes	laid by the virgin queen	laid by the mated queen	7	Transcriptome	*Apis mellifera*	[78]
embryos	24 h, 48 h, 72 h	——	——	laid by the mated queen	9	Transcriptome	*Apis cerana*	[84]
larva	2 d and 4 d	——	laid by the mated queen	worker, queen	36	Transcriptome	*Apis mellifera*	[79]
pupa	4 d (worker and drone), 3 d (queen)	brain	drone	worker, queen	9	Transcriptome	*Apis mellifera*	[80]
adult	Immediately after eclosion	residues (no head, thorax, and abdomen)	haploid drone, diploid drone	——	4	Transcriptome	*Apis cerana*	[77]
adult	——	antenna	sexually matured drone	time-trained forager	14	Transcriptome	*Apis mellifera*	[81]
adult	——	antenna	sexually matured drone	forager	——	Proteome	*Apis mellifera*	[82]
adult	——	antenna	sexually matured drone	forager, queen	——	Proteome	*Apis mellifera*	[83]
